# Weight and mid-upper arm circumference gain velocities during treatment of young children with severe acute malnutrition, a prospective study in Uganda

**DOI:** 10.1186/s40795-021-00428-0

**Published:** 2021-06-18

**Authors:** Jolly G. K. Kamugisha, Betty Lanyero, Nicolette Nabukeera-Barungi, Harriet Nambuya-Lakor, Christian Ritz, Christian Mølgaard, Kim F. Michaelsen, André Briend, Ezekiel Mupere, Henrik Friis, Benedikte Grenov

**Affiliations:** 1 Mwanamugimu Nutrition Unit, Department of Pediatrics, Mulago National Referral Hospital, P.O. Box 7051, Kampala, Uganda; 2 Department of Nutrition, Exercise and Sports, University of Copenhagen, 1958 Frederiksberg C, Denmark; 3 Department of Pediatrics and Child Health, College of Health Sciences, Makerere University, P.O. Box 7072, Kampala, Uganda; 4 Department of Pediatrics, Jinja Regional Referral Hospital, Jinja, Uganda; 5 Center for Child Health Research, Faculty of Medicine and Health Technology, Tampere University, Arvo building, Arvo Ylpön katu 34, FIN-33014 Tampere University, Tampere, Finland

**Keywords:** Severe acute malnutrition, Edema, Weight gain velocity, MUAC gain velocity, Children, Uganda

## Abstract

**Background:**

Weight gain is routinely monitored to assess hydration and growth during treatment of children with complicated severe acute malnutrition (SAM). However, changes in weight and mid-upper arm circumference (MUAC) gain velocities over time are scarcely described. We assessed weight and MUAC gain velocities in 6–59 mo-old children with complicated SAM by treatment phase and edema status.

**Methods:**

This was a prospective study, nested in a randomized/probiotic trial (ISRCTN16454889). Weight and MUAC gain velocities were assessed by treatment phase and edema at admission using linear mixed-effects models.

**Results:**

Among 400 children enrolled, the median (IQR) age was 15.0 (11.2;19.2) months, 58% were males, and 65% presented with edema. During inpatient therapeutic care (ITC), children with edema vs no edema at admission had negative weight gain velocity in the stabilization phase [differences at day 3 and 4 were − 11.26 (95% CI: − 20.73; − 1.79) g/kg/d and − 13.09 (95% CI: − 23.15; − 3.02) g/kg/d, respectively]. This gradually changed into positive weight gain velocity in transition and eventually peaked at 12 g/kg/d early in the rehabilitation phase, with no difference by edema status (*P* > 0.9). During outpatient therapeutic care (OTC), overall, weight gain velocity showed a decreasing trend over time (from 5 to 2 g/kg/d), [difference between edema and non-edema groups at week 2 was 2.1 (95% CI: 1.0;3.2) g/kg/d]. MUAC gain velocity results mirrored those of weight gain velocity [differences were − 2.30 (95% CI: − 3.6; − 0.97) mm/week at week 1 in ITC and 0.65 (95% CI: − 0.07;1.37) mm/week at week 2 in OTC].

**Conclusions:**

Weight and MUAC gain velocities among Ugandan children with complicated SAM showed an increasing trend during transition and early in the rehabilitation phase, and a decreasing trend thereafter, but, overall, catch-up growth was prolonged. Further research to establish specific cut-offs to assess weight and MUAC gain velocities during different periods of rehabilitation is needed.

**Supplementary Information:**

The online version contains supplementary material available at 10.1186/s40795-021-00428-0.

## Background

Severe acute malnutrition (SAM) affects an estimated 14.3 million children under-five globally [1] and contributes significantly to childhood morbidity and mortality [1, 2]. Children with complicated SAM are initially hospitalized for their treatment until stabilized, and are then discharged to outpatient therapeutic care (OTC), to continue treatment alongside those with uncomplicated SAM [3, 4]. The inpatient therapeutic care (ITC) process consists of an initial stabilization phase followed by a rehabilitation phase, with a transition period in between [3, 5].

During hospitalization, attained weight and weight gain velocity are monitored to track progress and identify children with failure to respond [5–8]. Attained weight is monitored daily. It is expected to remain stable for children without edema or to first drop down for those with edema in the stabilization phase, and to increase steadily during the rehabilitation phase [5, 9, 10]. Weight gain velocity is computed less often and during rehabilitation phase only. It is categorized as poor if < 5 g/kg/d, moderate if 5–10 g/kg/d and good if > 10 g/kg/d [11, 12].

Several studies have reported overall average weight gain velocities ranging from 1.8 to 8.9 g/kg/d [13–20] for children with uncomplicated SAM and from 7.2 to 15.6 g/kg/d in those with complicated SAM [10, 21, 22]. However, limited data exist regarding how weight gain velocity changes over time during treatment. An old study of hospitalized Jamaican children with SAM found variable individual mean weight gain velocities that were negative during the stabilization phase when the children were given a “maintenance” diet (91 kcal/kg/day) and positive during the rehabilitation phase when a catch-up diet (125 kcal/100 ml) was given [23]. Another study of hospitalized Jamaican children recovering from SAM showed that weight gain velocity decreases over time during the rehabilitation phase as children approach or reach a normal weight-for-length/height z-score (WHZ) [24]. However, these studies used old definitions of SAM, were conducted at the time when children with SAM were being treated as in-patients only and used locally prepared milk diets that provided different amounts and densities of energy and nutrients. Two recent studies of children recovering from uncomplicated SAM have shown that weight gain velocity decreases over time during OTC [25, 26], as expected. However, one of these studies did not distinguish between weight gain velocity by edema status [26] while the other involved only children with non-edematous SAM [25].

Some studies have looked at the role of edema in overall weight gain velocity in children with SAM but the results remain inconsistent [10, 19, 21]. Diop et al. [10] reported no differences in mean weight gain velocity in hospitalized Senegalese children with edematous SAM vs those with non-edematous SAM. In a Moroccan study, hospitalized children with marasmus (non-edematous SAM) had a higher mean weight gain velocity compared to their kwashiorkor (edematous SAM) counter parts [21]. In a Cameroonian study of children with uncomplicated SAM, lower mean weight gain velocities were found in those with non-edematous vs edematous SAM [19]. However, there is limited evidence regarding the role of edema in daily changes in weight gain velocity per treatment phase.

Mid-upper arm circumference (MUAC) has been accepted as an independent admission and discharge criteria [3], and is widely used for these purposes. An advantage is that it is less influenced by hydration status than body weight [27]. Several studies have found overall mean MUAC gain velocities ranging from 0.17 to 0.60 mm/d among children with uncomplicated SAM [13–16, 19, 25, 28]. Whilst some of these studies defined SAM using lower [15] or higher [13] MUAC cut-offs than the currrent WHO criteria [3], others excluded children with edema from the analysis [14, 15]. Further, in a study of Kenyan children with SAM and evidence of environmental enteric dysfunction (EED) [29], MUAC gain velocity from enrolment to day 28 was higher than from day 28 to day 56, suggesting a decreasing pattern over time. A few studies have compared MUAC growth by type of SAM but results are inconsistent [16, 19]. Trehan et al. [16] found a higher mean MUAC gain velocity among Malawian children with non-edematous SAM compared to those with edematous SAM. The Cameroonian study found higher mean MUAC gain velocity among children with marasmic kwashiorkor than those with marasmus or kwashiorkor [19].

In this study, we assessed the changes in weight and MUAC gain velocities among children with complicated SAM by treatment phase and edema at admission.

## Methods

### Study design, setting and population

This was a prospective cohort study among 400 children admitted with complicated SAM to Mwanamugimu Nutrition Unit (MNU), Mulago National Referral Hospital in Kampala, the capital city of Uganda between March 2014 and October 2015. The study was nested within a double blind randomized clinical trial (ProbiSAM), which investigated the effect of probiotics on diarrhoea in children with SAM (ISRCTN16454889) as detailed elsewhere [30]. Inclusion criteria were: children aged 6–59 months with complicated SAM defined as WHZ < − 3 or MUAC < 11.5 cm or bipedal pitting edema and having any medical complications and/or any integrated management of childhood illness danger sign(s), according to WHO criteria [3], with caregivers who provided written informed consent, and were willing to come back for follow-up. Exclusion criteria were: children in shock and or severe respiratory distress at admission (once stabilized, these children were considered for inclusion), admission weight less than 4.0 kg, obvious congenital anomalies and admission with SAM in the previous 6 months.

### Patient management

Patient management has been described in detail elsewhere [31, 32]. In brief, all patients in the study received standard treatment following the WHO guidelines [3] and the integrated management of acute malnutrition (IMAM) guidelines for Uganda [33]. The in-patient treatment was divided into two phases, phase 1 or stabilization phase and phase 2 or rehabilitation phase, with a transition stage in between.

In the initial stabilization phase, all patients received F-75 (Nutriset, Malaunay, France) at 100-130 ml/kg/day depending on the grade of edema and routine medical treatment usually intravenous ampicillin and gentamycin. For breastfed children, mothers were encouraged to breastfeed in-between the therapeutic feeds. All children with diarrhoea were given Rehydration Solution for the Malnourished (ReSoMal, Nutriset, Malaunay, France), according to their body weight and degree of dehydration. When children were ready for transition as indicated by return of appetite, edema subsiding to grade one or two and medical complications resolving, they were subjected to acceptance test for Ready-to-Use-Therapeutic Food (RUTF), Plumpy’nut® (Nutriset, Malaunay, France). Children who passed the acceptance test were gradually transitioned from F-75 to RUTF over 2–3 days while those who failed were given F-100 (Nutriset, Malaunay, France), prescribed to provide 100–135 kcal/kg/day. For children on F-100, RUTF was re-tried after 2–3 days. In the rehabilitation phase, children were given increasing amounts of RUTF or F-100 based on their weight and appetite and, were also introduced to *kitobeero*, a multi-mix and nutritious local dish. The feeds offered provided 150–200 kcal/kg/day.

When the children were clinically well, with good appetite and edema resolved to grade 1 or 2, they were discharged to OTC with RUTF, and together with their caregivers, they were appointed for follow-up every second week. Children continued to receive RUTF and were followed up to for a minimum of 8 weeks, and those who had not recovered by then were followed up to a maximum of 12 weeks.

### Study outcomes

The main study outcomes were weight and MUAC gain velocities during the different treatment phases in ITC and in OTC. Weight gain velocity expressed as g/kg/d was calculated in accordance with WHO [7] as follows:

$$ \left(\left(\mathrm{W}2-\mathrm{W}1\right)\times 1000/\mathrm{W}1\right)/\mathrm{t} $$

where W2 is the weight at current measurement and W1 is the weight at previous measurement and t is the number of days between measurements (for ITC, t = 1 and for OTC, t = 14, on average). MUAC gain velocity was defined as gain in mm per week and was calculated as follows:

$$ \left(\mathrm{M}2-\mathrm{M}1\right)\times 10/\left(\mathrm{t}/7\right) $$

where M2 is the MUAC (cm) at current measurement and M1 is the MUAC (cm) at the previous measurement, t is the number of days between measurements (for ITC, t = 7 and for OTC, t = 14, on average).

Other outcomes included duration of stay in stabilization phase, transition and rehabilitation phase in ITC and OTC, duration of hospitalization, proportion of children recovered and not recovered. Proportions of children recovered and not recovered were defined as (number of children who reached or did not reach nutritional recovery after 12 weeks in OTC/total number of children discharged to OTC) × 100. Nutritional recovery was defined as reaching the WHO (2013) discharge criteria of WHZ ≥ − 2 or MUAC ≥12.5 cm and no oedema for 2 consecutive weeks and clinically well and alert. The same anthropometric criteria used to diagnose a child as SAM was used to decide whether the child had reached nutritional recovery. Further, if a child met both anthropometric criteria or had edema at admission, the child was discharged based on WHZ ≥ − 2. A child was only considered self-discharged if they did not return to the ward or the OTC site before the week 12 visit.

### Data collection procedures

A case report form (CRF) was used to systematically document all data obtained from the caregiver and patient examination findings. At admission, data was collected on the child’s socio-demographic information, breastfeeding and medical history, as well as presenting symptoms. Caregivers were asked to grade the severity of illness using a Visual Analogue Scale (VAS) and about household information where the child had lived two months prior to admission. Data on maternal age, marital status, education level and occupation were collected, if available. Food insecurity was assessed using the validated Household Food Insecurity Access Scale (HFIAS) [34]. A full physical examination including grade of edema, dehydration status, skin changes and vital signs was performed by a study pediatrician. On admission, weekly during hospitalization, at discharge and during OTC visits, child’s anthropometric measurements (weight, MUAC and length/ height) were taken in accordance with the WHO guidelines [3]. The same trained study nutritionists took measurements at different time points during ITC and OTC. Child’s body weight was measured (naked and without shoes) in triplicate using a digital scale (Seca 813, Hamburg, Germany) to the nearest 100 g. Triple MUAC measurements were taken on the child’s left arm without clothes, using standard non-elastic color coded tapes for under 5-year-old children (Child 11.5 red/pac-50, UNICEF) to the nearest 1 mm. Length/ height was measured in triplicate using an infant length board (Infant/ Child Shorr-Board®, Maryland, USA) to the nearest 1 mm (supine length was measured for children less than 24 months of age). Body weight was further measured once daily at 7:00 AM before the 8:00 AM feed during hospitalization. On admission, maternal body weight was measured in triplicate using a digital scale (Seca 813, Hamburg, Germany) to the nearest 100 g. Height was measured in triplicate using an adult height board (adult Shorr-Board®, Maryland, USA) to the nearest 1 mm. Before measurements were taken, mothers were asked to remove shoes and any extra clothes. For measurements taken in triplicate, the average was considered. WHZ and height/length-for-age z-score (HAZ) were computed using WHO Anthro version 3.2.2 [15]. Maternal body mass index (BMI) expressed in kg/m^2^ was calculated as weight in kg divided by the square of the height in meters [35]. Throughout the study period, clinical evaluation of patients was performed by the same study pediatricians. Likewise, the same study nutritionists were responsible for conducting nutritional evaluation. This included monitoring of vital signs, grade of edema, appetite, anthropometry, type and amount of feeding regimens given daily during ITC and bi-weekly during OTC visits. Based on this, decisions regarding management of the patients were jointly made.

### Laboratory tests

Four [4] ml of blood was drawn by venipuncture and placed into heparinized vacutainer tubes (Becton Dickinson, Franklin lakes, NJ USA) by the study doctor or nurse. Complete blood counts were analyzed using a coulter counter at the Uganda cancer institute laboratory. HIV serological testing was done at MNU side lab using rapid tests (Determine HIV-1/2 [Abbot Laboratories USA], and positive samples were confirmed with HIV-1/2 Stat-Pak Dipstick Assay kit. HIV DNA/PCR test was done at the hospital’s HIV clinic for all children aged below 18 months with a positive serology test. Plasma was obtained by centrifuging at 1300-2200G for 10 min, then stored at − 80 °C at Immunology Laboratory, Mulago Hospital until shipped on dry ice to the Department of Nutrition, Exercise and Sports, University of Copenhagen, Denmark. Plasma C-reactive Protein (CRP) was measured by high sensitive kit on an ABK Pentra 400 (Horiba, Montpellier, France).

### Data handling and statistical analysis

All data were double entered into Epidata Version3.1 (Odense, Denmark) and analyzed using R version 3.5.1 (R Core Team, 2017), with the extension packages plyr, dplyr, lme4, multcomp, car and ggplot2. Missing daily weight data were imputed using the “linear interpolation” approach, whereby a missing value was linearly interpolated by using values before and after the missing one [36]. Daily weight gain data were organized according to days of follow-up per treatment phase depending on the feeds received and daily energy intake. For ITC, weight gain velocity is presented based on 8, 4 and 5 days in stabilization, transition and rehabilitation phases, respectively. For OTC, it is presented based on 8 and 12 weeks. MUAC gain velocity is presented weekly and bi-weekly for ITC and OTC, respectively. The terms “rehabilitation phase” and “OTC” refer to the rehabilitation phase in ITC and OTC, respectively.

Baseline child, maternal and household characteristics based on continuous variables were presented as means ± standard deviations (SD) or median [interquartile range (IQR)], and categorical variables were presented as percentages (n). To evaluate whether median duration of treatment phases differed by edema at admission, we used Mann-Whitney U Test.

Linear mixed-effects models (with restricted maximum likelihood estimation) were used to investigate the changes in weight and MUAC gain velocities between time points during ITC and OTC, adjusted for a priori potential confounders (age, sex, HIV status). The models included child-specific random intercepts and robust SEs were used. Because weight gain differs by edema at admission [5, 7], the models also included interaction terms between time of follow-up and edema at admission. To obtain estimate changes (b-coefficients) due to exposure between time points, unadjusted and age-sex adjusted models were fitted separately for stabilization, transition, rehabilitation and OTC. All models were checked based on residuals and predicted random effects using residuals plots and normal probability plots. *P*-values below 0.05 were considered statistically significant.

## Results

**Fig. 1:**
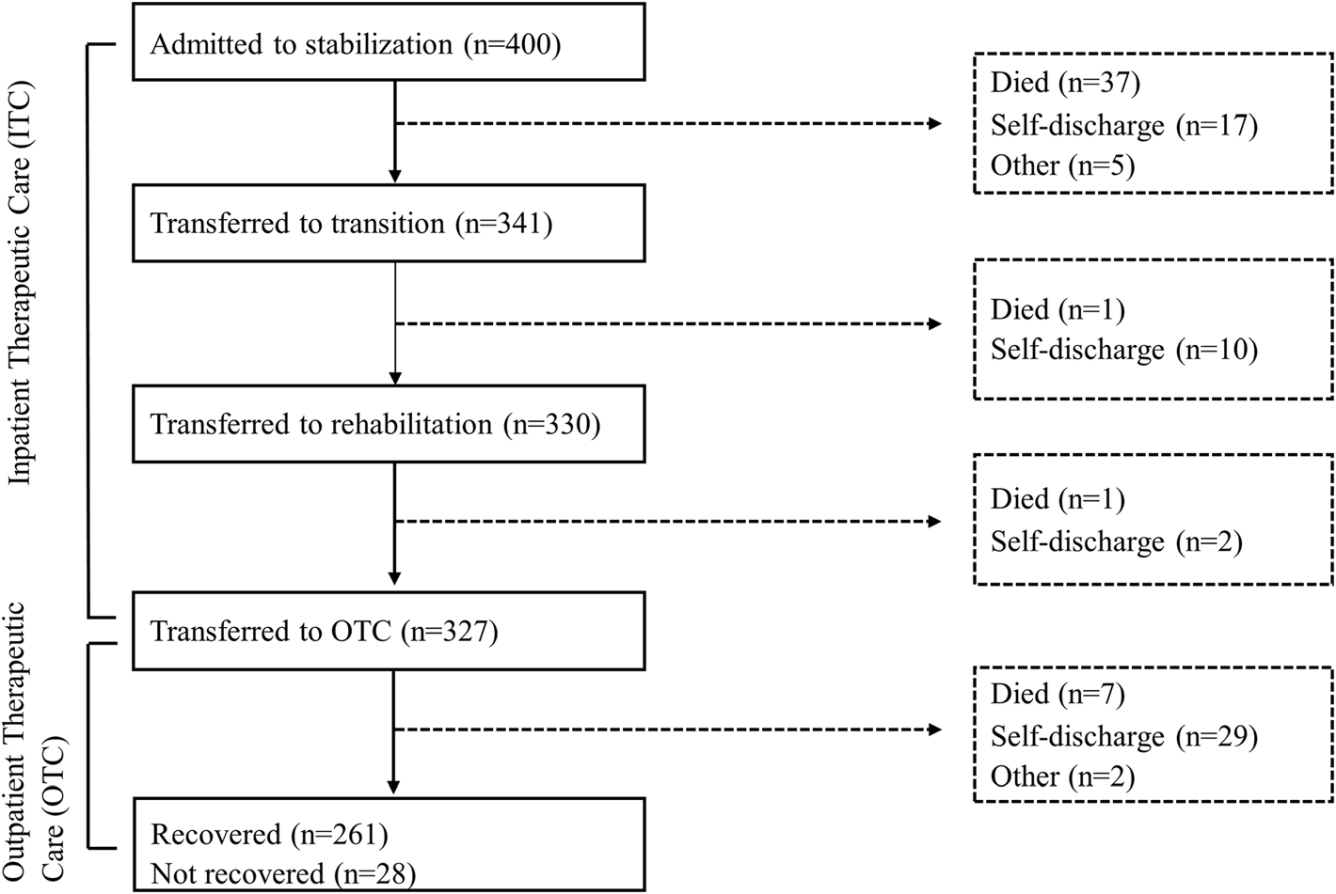
Flow chart of study participants

Among 400 children with SAM enrolled into the parent ProbiSAM trial, the median (IQR) age was 15.0 (11.2; 19.2) months, 58% were males, and 65% presented with edema (Table 1). Of these 400, 341 (85%) progressed from stabilization phase to transition and 330 (83%) progressed to rehabilitation phase. Eventually, 327 (82%) were discharged and transferred to OTC (Fig. 1). Of the discharged children, 261 (80%) reached nutritional recovery, majority of whom (77%) recovered within 8 weeks of follow-up.

**Table 1:** Baseline characteristics of 400 children admitted with severe acute malnutrition ^a^

Characteristic	***N***	
**Child**		
Age (months)	400	15.0 (11.2; 19.2)
Female sex	400	170 (42%)
Weight-for-length/height Z-score	387	−2.9 (− 3.7; − 1.5)
Length/height-for-age Z-score	387	− 3.1 ± 1.4
Currently breastfeeding	374	54 (14%)
Edema	399	
No		138 (35%)
Yes		261 (65%)
Diarrhoea	400	241 (60%)
Dehydration	395	10 (3%)
Tuberculosis suspected	400	76 (19%)
Pneumonia	400	68 (17%)
HIV status	368	
Positive		43 (12%)
Negative, exposed		72 (20%)
Negative		253 (69%)
Serum C-reactive protein (mg/L)	352	
< 10		134 (38%)
≥ 10		218 (62%)
Hemoglobin (g/dL)	298	8.9 (7.8; 10.1)
Severity of illness ^b^	399	6.0 (5.0; 7.0)
**Mother**		
Age	356	24.0 (21.0; 28.0)
BMI (kg/m^2^)	334	22.7 (20.9; 25.3)
Education	385	
Primary		181 (47%)
Secondary or higher		153 (40%)
HIV positive	340	109 (32%)
**Household**	389	
Household food insecurity access scale score ^c^		5.8 ± 7.0

^a^ Data are number of children with data (*N*), and median (interquartile range) or mean ± standard deviation or number (%)

^b^ Evaluated by the caregiver on a visual analogue scale from 0 to 10, where 0 = perfectly healthy, 10 = as sick as I can imagine

^c^ A measure of food insecurity in the household in the past four weeks on a scale from 0 to 27; the higher the score, the more food insecure the household has been

The overall median (IQR) duration of stay in the stabilization, transition and rehabilitation phases of ITC and, hospitalization was 7 (5; 11), 3 (2; 5), 4 (3; 5) and 17 (12; 22) days, respectively (data not shown). In OTC, it was 56 (56; 58) days, but was pre-defined by the parent trial. As shown in Supplemental Table 1**,** the median (IQR) duration of stay was longer in the stabilization phase [8 (5; 12) days vs 7 (4; 10) days, *P* < 0.001] but shorter in the transition phase [3 (2; 5) days vs 3 (2; 6) days, *P* = 0.026] among children with edema compared to those without edema at admission, whereas there were no differences in the rehabilitation phase in ITC, in hospitalization and in OTC, *P* > 0.08.

**Fig. 2:**
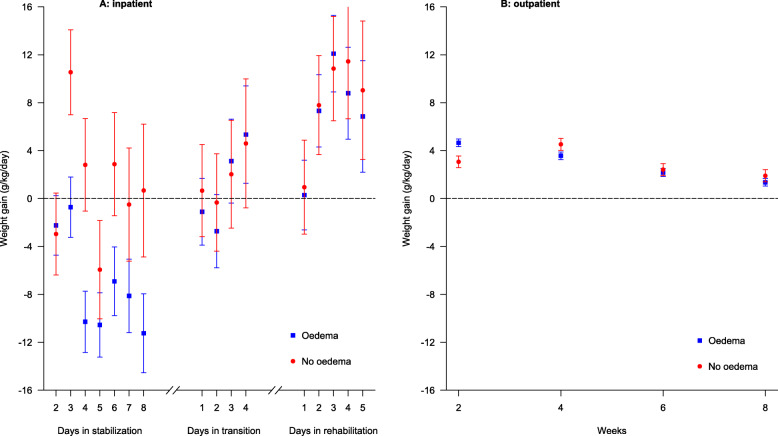
Weight gain velocity presented as mean (standard error) g/kg/d for children with severe acute malnutrition by edema at admission and treatment phase

As seen in Fig. 2**(panel A)**, during ITC, children with edema had negative daily weight gain velocities throughout the stabilization phase and the initial 2 days of transition. This was followed by positive weight gain velocities, peaking at 12 g/kg/d three days into the rehabilitation phase. Among children without edema the mean weight gain velocities were variable, but mainly positive during the stabilization phase, but were then similar to what was seen for children with edema during the transition and rehabilitation phases. During OTC (Fig. 2**, panel B**), among children with edema at admission, the mean weight gain velocities declined from around 5 g/kg/d during the first 2 weeks to around 2 g/kg/d from 6 to 8 weeks. For children without edema at admission, the weight gain velocities were slightly lower during the first 2 weeks and peaked after 4 weeks.

The weight gain velocities were, however, highly variable for each child over time and between children at a given time as shown in Supplemental Fig. 1. The spaghetti plot shows changes in weight gain velocities by treatment phase from four children i.e., a 16 months old non-breastfed, HIV positive child (child 1), a 51 months old non-breastfed, HIV negative child (child 2), both with edema and diarrhoea at admission, as well as 11 months old breastfed child without diarrhoea at admission (child 3) and a 23 months old non-breastfed child (child 4) with diarrhoea at admission, both HIV exposed and without edema at admission.

In Table 2, the differences in mean daily weight gain velocities during ITC and OTC for edematous compared to non-edematous children are given. In the stabilization phase, there was no difference at day 2, whereas weight gain velocity in children admitted with edema was 11–13 g/kg/d lower at day 3 and 4 compared to children admitted without edema. From day 5 to 8, the point estimates were consistently lower among edematous children, but the differences in weight gain velocity were not significant. Yet, there was no significant interaction between edema status at admission and time (*P* = 0.315).

**Table 2:** Differences in weight gain velocities for children admitted with edematous compared to non-edematous severe acute malnutrition during the stabilization, transition and rehabilitation phases of inpatient therapeutic care, and during outpatient therapeutic care†

			Unadjusted	Age-sex adjusted	Edema x time
	**Time**	**n**	**b (95%CI)**	**b (95%CI)**	***P*-value**
**Stabilization**	Day 2	360	0.72 (−8.43; 9.87)	0.75 (− 8.45; 9.95)	0.315
	Day 3	351	− 11.27 (−20.68; − 1.85)*	− 11.26 (− 20.73; − 1.79)*	
	Day 4	319	− 13.10 (− 23.12; − 3.08)*	− 13.09 (− 23.15; − 3.02)*	
	Day 5	283	−4.61 (− 15.22; 6.00)	− 4.63 (− 15.27; 6.02)	
	Day 6	250	− 9.78 (− 20.96; 1.40)	− 9.82 (− 21.04; 1.40)	
	Day 7	209	−7.63 (− 19.80; 4.55)	−7.66 (− 19.87; 4.55)	
	Day 8	179	− 11.91 (− 25.85; 2.02)	−12.02 (− 26.00; 1.96)	
**Transition**	Day 1	302	−1.77 (−9.81; 6.29)	− 1.70 (− 9.85; 6.45)	0.918
	Day 2	258	− 2.40 (− 11.01; 6.23)	− 2.33 (− 11.05; 6.38)	
	Day 3	201	1.10 (− 8.57; 10.77)	1.14 (− 8.58; 10.86)	
	Day 4	146	0.74 (−10.70; 12.18)	0.79 (−10.71; 12.30)	
**Rehabilitation**	Day 1	282	−0.65 (−9.12; 7.82)	− 0.21 (−8.77; 8.34)	0.995
	Day 2	258	−0.46 (−9.36; 8.42)	− 0.11 (− 9.057; 8.84)	
	Day 3	231	1.25 (−8.14; 10.63)	1.57 (−7.86; 11.00)	
	Day 4	171	−2.65 (− 13.31; 8.01)	−2.31 (− 13.01; 8.39)	
	Day 5	117	−2.19 (−15.06; 10.69)	−1.90 (− 14.80; 11.01)	
**Outpatient**	Week 2	295	1.90 (0.84; 2.95)*	2.09 (1.03; 3.15) *	0.002
	Week 4	285	−0.70 (−1.77; 0.37)	−0.49 (− 1.57; 0.58)	
	Week 6	284	−0.64 (−1.71; 0.43)	− 0.44 (− 1.52; 0.64)	
	Week 8	284	−0.50 (−1.58; 0.58)	− 0.31 (− 1.39; 0.77)	
	Week 10	27	−0.43 (−3.40; 2.94)	− 0.31 (− 3.67; 3.05)	
	Week 12	19	2.55 (−1.47; 6.58)	2.75 (−1.27; 6.76)	

**†** Data are number (*n*), regression coefficients (b) and 95% confidence intervals (CI). *P < 0.05. During ITC, numbers are varying each day because children are transferred from one phase to another based on condition. During OTC, there are small variations in numbers analyzed (week 2–8) because of missing data. After 8 weeks, the numbers are very few because follow-up was ended at 8 weeks for those who had recovered

The estimates did not change after age and sex adjustments. Also, further adjustment for HIV status did not change the estimates by much, hence it was not included in the final models. There were no differences in weight gain velocities by edema at admission in the transition and rehabilitation phases. In OTC, a 2 g/kg/d higher weight gain velocity was seen in edematous vs non-edematous children up to 2 weeks, whereas no differences were seen afterwards.

**Fig. 3:**
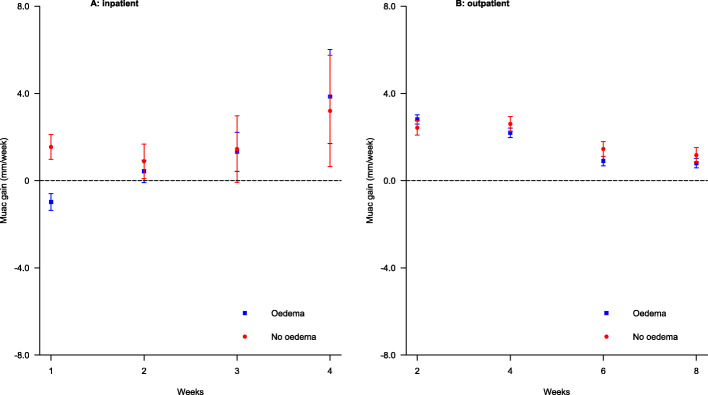
Mid-upper arm circumference (MUAC) gain velocity presented as mean (standard error) mm/week for children with severe acute malnutrition who were discharged in ≤ 4 weeks and those who recovered in ≤ 8 weeks by edema at admission and treatment phase

Figure 3 shows changes in mean MUAC gain velocities for children hospitalized for 4 weeks or less and those who recovered within 8 weeks in OTC by treatment phase and edema at admission. During ITC (Fig. 3**, panel A**), among children with edema, the mean MUAC gain velocity was negative during the first 1 week but then increased steadily, peaking at around 4 mm/week after 4 weeks. Among children without edema, the mean MUAC gain velocities declined from around 2 to around 1 mm/week after 2 weeks, but increased to around 3.5 mm/week after 4 weeks, similar to what was seen for children with edema. During OTC (Fig. 3**, panel B**), mean MUAC gain velocities declined from around 3 mm/week after 2 weeks to < 1 mm/week from 6 to 8 weeks, irrespective of edema at admission.

Table 3 shows the differences in mean weekly and bi-weekly MUAC gain velocities during ITC and OTC, respectively, for edematous compared to non-edematous children.

**Table 3:** Differences in mid-upper arm circumference (MUAC) gain velocities for children admitted with edematous compared to non-edematous severe acute malnutrition during inpatient therapeutic care and outpatient therapeutic care†

			Unadjusted	Age-sex adjusted	Edema x time
	**Time**	***n***	**b (95%CI)**	**b (95%CI)**	***P*-value**
**Inpatient**	Week 1	335	−2.44 (−3.74; −1.14)*	− 2.30 (− 3.6; − 0.97)*	0.298
	Week 2	186	−1.01 (− 2.77; 0.75)	−0.91 (− 2.68; 0.86)	
	Week 3	69	0.80 (−2.13; 3.73)	0.86 (− 2.08; 3.79)	
	Week 4	26	0.23 (−2.13; 3.73)	0.22 (−4.18; 4.63)	
	Week 5	15	0.15 (−5.65; 5.94)	0.10 (−5.70; 5.89)	
**Outpatient**	Week 2	295	0.57 (−0.15; 1.28)	0.65 (− 0.07; 1.37)	0.091
	Week 4	285	−0.53 (−1.26; 0.20)	− 0.45 (− 1.18; 0.29)	
	Week 6	284	−0.49 (−1.22; 0.24)	− 0.41 (− 1.14; 0.33)	
	Week 8	284	−0.43 (−1.17; 0.31)	− 0.35 (− 1.09; 0.39)	
	Week 10	27	1.63 (−0.66; 3.93)	1.68 (−0.61; 3.98)	
	Week 12	19	−0.58 (−3.32; 2.15)	− 0.53 (−3.27; 2.21)	

**†** Data are number (*n*), regression coefficients (b) and 95% confidence intervals (CI). *P < 0.001. During ITC, numbers are varying each week because children are transferred from one phase to another based on condition. During OTC, there are small variations in numbers analyzed (week 2–8) because of missing data. After 8 weeks, the numbers are very few because follow-up was ended at 8 weeks for those who had recovered

In ITC, a 2 mm/week lower MUAC gain velocity was seen in children with edema vs without edema at admission up to 1 week, whereas no differences were seen afterwards. In OTC, there was a marginally higher mean difference in MUAC gain velocities after 2 weeks and similar but lower mean differences after 4, 6, and 8 weeks in edematous vs non-edematous children. These differences were however not statistically significant.

For MUAC and weight gain velocities for children who stayed longer than 4 weeks in ITC and 8 weeks in OTC, supplemental Figs. 2 and 3 are available.

## Discussion

### Variability in weight gain velocity

The striking variability in daily weight gain velocity, particularly in the stabilization phase, may be due to considerable biological variation as well as technical error. Diarrhoea and vomiting are common among children with SAM and cause dehydration [3, 37, 38]. In this study, 60% of the children had diarrhea while 43% had vomiting at admission and others developed diarrhoea during hospitalization. Hydration status and substances in the digestive tract can lead to variations in weight within short periods of time, because of changes in total body water [27]. There is limited published literature describing day to day changes in weight gain velocity among acutely ill hospitalized children with SAM. A study of Bangladeshi children with SAM and cholera found an average weight gain of around 9 to 11% after 24 to 72 h of inpatient treatment [39]. In another study of Rwandan children with gastroenteritis (not severely malnourished), Pringle et al. [40] reported a median percentage weight gain of 4.8% from admission to discharge, and over 10% weight gain in 28% of the children. In a study of Kenyan children (24% of whom had SAM) with clinical signs of dehydration, body weight measurements at admission and after 48 h indicated a significant change in weight following rehydration [27]. The same study found that 75 and 25% of the children gained and lost weight, respectively, and the authors attributed the observed weight changes to changes in body water and not tissue deposition [27]. A child with SAM is unlikely to put on tissue during the initial phase, as the starter feed used for stabilization (F-75) is only for maintenance of basic physiological processes [11], and nearly all weight change can be related to water movements due to edema, fluid shifting (activation of Na-K pumps), diarrhoea or rehydration fluids. It is not uncommon to see a dehydrated child with diarrhoea who has lost 5% of its body weight within a few days [27, 40, 41]. This corresponds to a change of 50 g/kg. When these children are rehydrated, they get a weight gain of the same amplitude. These results are also consistent with findings of the Kenyan study described above, showing that it may be possible that a child is categorized as SAM due to dehydration, but, after rehydration, the child may be classified as MAM [27]. Therefore, re-assessment of anthropometry and re-categorization of nutritional status should be considered for better case management. However, during the rehabilitation phase, significantly smaller weight gains of around 10 to 15 g/kg/d have been observed [10, 21]. Further, in this study, weighing scales with a low precision (100 g) were used. Also, daily single weight measurements, except for weekly triple measurements, were taken during hospitalization. These factors are likely to have contributed to the high day-to-day variation in weight gain velocity. However, a much less variation in weight gain velocity was seen during outpatient treatment because there is less biological variation (less vomiting, diarrhoea and fever), and the estimate is based on the mean of triple measurements taken over 2-week periods. Weight gain velocity estimate requires two measurements and as such, it involves two measurement errors. Moreover, measurement errors are greater with single compared to repeated measurements [42]. Furthermore, weight gain velocities were variable among the children, suggesting a variable response to treatment.

### Changes in weight gain velocities

As expected, a mean negative weight gain velocity was found among children with edema compared to those without edema at admission in the stabilization phase. This is related to weight loss due to edema resolution during initial feeding with a low-calorie, specialized formula, F-75. This finding is consistent with those of an old study of hospitalized young Jamaican children with SAM, indicating that individual mean weight gain velocity during the acute phase when children were receiving a “maintenance” diet varied between − 10.9 to − 7.3 g/kg/d [23]. The authors concluded that the observed negative weight gain velocity was explained by a negative energy balance [23]. It is important to note that the children in the Jamaican study had no diarrhoea unlike in the current study. Another study in the Democratic Republic of Congo observed that in the first 7 days of hospitalization, attained weight first dropped among children with edematous SAM, while it remained stable among children non-edematous SAM, but then it increased from 7 to 21 days in both types of SAM [9]. Although we found no interaction between time of follow-up and edema at admission, there was a lower difference in mean weight gain velocity at days 3 and 4 among children with edema vs no edema. Nonetheless, we had expected to see large differences in the first few days, and then declining differences. The reason we did not see this is probably that those responding were transitioned, thus, those remaining in stabilization were those who responded with a delay.

A positive weight gain velocity was observed during transition and rehabilitation phases with no differences between edematous and non- edematous children (P for interaction > 0.9). Weight gain velocity ranged from around 2 g/kg/d to around 12 g/kg/d from day 1 to day 3, and from around 12 g/kg/d to around 8 g/kg/d from day 3 to day 5 of the rehabilitation phase. These results indicate that there is accelerated catch-up growth in the first few days of rehabilitation phase and later on, the catch-up growth is gradual, as expected. This is explained by the change in diet from F-75 to RUTF or F-100, which are higher energy and protein “catch-up” formulae intended to rebuild wasted tissues [3, 11]. Our findings are in agreement with those of Spady et al. [23] who found mean weight gain velocities ranging from 3.2 to 21.5 g/kg/d among hospitalized children with SAM who were receiving a high energy diet for catch-up growth during the rehabilitation phase.

The observed decreasing trend of weight gain velocity during OTC is not surprising since during recovery from SAM, a rapid weight gain velocity is seen initially when children have low WHZ, but then it falls to 1–2 g/kg/d when a normal WHZ is approached [43, 44]. Studies have shown that WHZ is inversely correlated with weight gain velocity in OTC [13, 45]. This finding is consistent with those of recent studies of young children recovering from uncomplicated SAM which found a rapid decrease in weight gain velocity after the initial weeks of treatment [25, 26]. However, the weight gain velocity from 4 weeks onwards was higher in the current study than in the study by Kangas et al. [25]. These results suggest that children with complicated SAM have a prolonged catch-up growth and indicate that catch-up may depend on how sick the child has been. Further, we found higher weight gain velocity over the first 2 weeks in children with compared to those without edema at admission (*P* = 0.002 for interaction between edema and time), suggesting accelerated catch-up growth in those with edema at admission. Kwashiorkor is often considered an acute dysadaptation; after disappearance of edema, children achieve accelerated growth and later on it is gradual. In contrast, marasmus is believed to be a chronic reductive adaptation, thus, children have a gradual onset of catch up. These findings are also consistent with those of a study of Indian children admitted with SAM, showing that those with non-edematous SAM started gaining weight earlier than their edematous counter parts: mean (SD) 4.4 (3.9) days vs 11.4 [7] days [22].

A constant weight gain velocity expressed in g/kg/d reflects an exponential growth, which is never seen in multicellular organisms. Hence, it is bound to decrease over time and it is not surprising that we see this in all studies including this one. This has important implications when weight gain velocity is used to assess catch-up growth as part of a national or international health policy. The WHO guidelines [3, 11] mention just one set of criteria to assess weight gain velocity, which may be valid only at the beginning of the rehabilitation phase, but no more after a few weeks of treatment. This highlights the need for considering use of different cut-offs to assess weight gain velocity at different stages of catch-up growth.

### Changes in MUAC gain velocities

During ITC, a negative trend of MUAC was seen among children with edema at admission, whereas a declining trend was seen among children without edema at admission in the early period. This is similar to what was observed for weight gain velocity during the stabilization phase and is mainly explained by edema resolution. From 2 weeks onwards (during catch-up), MUAC gain velocity is positive and shows an increasing trend in both groups, with no difference by edema at admission, similar to what was observed for weight gain velocity during transition and rehabilitation phases. During OTC, the observed decreasing trend of MUAC gain velocity over time mirrors that of weight gain velocity, suggesting a decreasing trend in MUAC growth. This finding is consistent with those of other studies [26, 29]. A recent study in the Gambia of children with uncomplicated SAM demonstrated a positive correlation between percentage weight gain and percentage MUAC gain [14]. Similarities between attained weight changes and MUAC changes at each follow-up visit in OTC have also been reported in a study of Indian children [46] and another study that compared data for children with uncomplicated SAM from two countries in Africa (Malawi, Ethiopia) and one in Asia (Bangladesh) [47], plus a large study in Burkina Faso [13]. A study in Malawi also found that changes in mean MUAC are directly related to changes in mean body weight during treatment of moderately malnourished children [48].

In the current study, we found less variability in MUAC gain velocity than seen in weight gain velocity. This finding is consistent with those of Mwangome et al. [27] showing that MUAC is less affected by hydration status than weight. There are currently no internationally agreed standards relating to use of MUAC as a tool for monitoring response to nutritional rehabilitation. However, these results provide evidence regarding the potential of MUAC gain velocity in tracking catch-up growth among children recovering from SAM.

Overall, these results suggest that recovery continues throughout treatment in OTC, similar to findings by Kangas et al. [25]. This observation has important clinical implications as SAM affects different body components that may take a while to recover. A study based on the same population as ours found that thymus gland does not recover after 8 weeks of treatment in OTC, despite recovery of MUAC [49]. This could partly explain the high post-discharge mortality reported in many studies of children with SAM. These results suggest that OTC follow-up should not be ended based on recovery of simple anthropometric parameters as per the current WHO guidelines [3].

### Strengths and limitations

This is among the few studies that describe the changes in both weight and MUAC gain velocities among children with complicated SAM by treatment phase and edema at admission. The limitations include measurement of weight using a weighing scale with a low precision (100 g) and taking single measurements on most days. However, body weight measurements were taken at the same time (i.e. one hour after feeding) by trained nutritionists and, daily single measurements were replaced with means of triple weekly measurements when available. Moreover, missing data could have introduced bias, but we used linear interpolation to impute the missing values for daily weight data using values before and after the “missing value” in order to yield a more complete data set [36].

## Conclusions

Despite the huge variability in weight gain velocity in the stabilization phase, we find evidence of an increasing trend during transition and early in the rehabilitation phase in ITC followed by a decreasing trend, but, overall, there is elongated catch-up growth among Ugandan children with complicated SAM. The study also reveals that children with edematous SAM have a higher weight gain velocity over the first 2 weeks in OTC compared to those with non-edematous SAM, which is suggestive of accelerated catch-up growth among those with edema at admission. Further, changes in MUAC gain velocity over time mirrored those of weight gain velocity but with less variability. The results support the WHO guidelines [7, 11] that require that changes in weight be monitored closely during stabilization to assess water movements for clinical management purposes. To minimize relapse to SAM and subsequent mortality, policy makers and practitioners should augment the current WHO discharge criteria [3] by considering a longer period of OTC follow-up. Moreover, the finding that higher weight gain velocities are expected initially in the rehabilitation phase highlights the need for different cut-off values for different periods of rehabilitation but this needs to be studied. Future studies should also consider MUAC gain in assessing growth in clinical settings because it is less affected by short-term changes that affect weight and hydration status (vomiting, drinking, eating, passing urine or diarrheal stools), and could potentially be used alongside weight gain, as a marker of growth, especially in the rehabilitation phase.

## Supplementary Information

**Additional file 1: Supplemental Table 1.** Duration of stay and outcome of children admitted with severe acute malnutrition by edema at admission ^a^.

**Additional file 2: Supplemental Figure 1.** Spaghetti plot showing variability in weight gain velocity among four selected children with severe acute malnutrition by treatment phase. Data are presented as absolute values (g/kg/d). Child 1, 16 months old non-breastfed, HIV positive with oedema and diarrhoea at admission; child 2, 51 months old, non-breastfed, HIV negative and, with oedema and diarrhoea at admission; child 3, 11 months old, breastfed, HIV exposed and, with no diarrhoea or oedema at admission; child 4, 23 months old, non-breastfed, HIV exposed, with diarrhoea at admission and no oedema at admission.

**Additional file 3: Supplemental Figure 2.** Mid-upper arm circumference (MUAC) gain velocity presented as mean (standard error) mm/week for children with severe acute malnutrition who stayed longer than 4 weeks in inpatient therapeutic care and 8 weeks in outpatient therapeutic care by edema at admission and treatment phase.

**Additional file 4: Supplemental Figure 3.** Weight gain velocity presented as mean (standard error) g/kg/d for children with severe acute malnutrition who stayed longer than 8 weeks in outpatient therapeutic care by edema at admission.

## Data Availability

The datasets used and/or analysed during the current study are available from the corresponding author on reasonable request.

## Abbreviations

BMI: Body mass index
CI: Confidence interval
EED: Environmental enteric dysfunction
HAZ: Height/length-for-age z-score
HFIAS: Household Food Insecurity Access Scale
HIV: Human Immunodeficiency Virus
IMAM: Integrated management of acute malnutrition
ITC: Inpatient therapeutic care
MNU: Mwanamugimu Nutrition Unit
MUAC: Mid-upper arm circumference
NDA: Uganda National Drug Authority
OTC: Outpatient therapeutic care
ReSoMal: Rehydration Solution for the Malnourished
RUTF: Ready-to-Use Therapeutic Food
SAM: Severe acute malnutrition
SOMREC: Makerere University School of Medicine Research Ethics Committee
UNCST: Uganda National Council for Science and Technology
VAS: Visual analogue scale
CRF: Case report form
WHO: World Health Organization
WHZ: Weight-for-length/height z-score
