# Supplementary material for: Weight and mid-upper arm circumference gain velocities during treatment of young children with severe acute malnutrition, a prospective study in Uganda
Source: BMC Nutr. 2021 Jun 18;7:26. doi: 10.1186/s40795-021-00428-0 (PMC8212498; doi:10.1186/s40795-021-00428-0)
Supplement: Supplementary file 1 — Additional file 1: Supplemental Table 1. Duration of stay and outcome of children admitted with severe acute malnutrition by edema at admission a. [file 40795_2021_428_MOESM1_ESM.docx]

| **Supplemental Table 1:** Duration of stay and outcome of children admitted with severe acute malnutrition by edema at admission ^a^ | | | |
| --- | --- | --- | --- |
| **Characteristic** | **Edematous SAM (N = 261)** | **Non-edematous SAM (N= 138)** | **P-value** |
| Duration of stay in stabilization phase | 8 (5; 12) | 7 (4; 10) | < 0.001 |
| Duration of stay in transition | 3 (2; 5) | 3 (2; 6) | 0.026 |
| Duration of stay in rehabilitation phase ^b^ | 4 (3; 5) | 4 (3; 6) | 0.088 |
| Duration of hospitalization | 17 (13; 23) | 17 (12; 22) | 0.381 |
| Duration of stay in outpatient therapeutic care | 56 (56; 58) | 57 (56; 62) | 0.197 |
| Recovered | 188 (72%) | 73 (53%) | < 0.001 |
| Not recovered | 21 (8%) | 26 (19%) | 0.001 |
| Self-discharge | 27 (10%) | 30 (22%) | 0.002 |
| Died | 28 (11%) | 19 (14%) | 0.370 |
|  |  |  |  |
|  |  |  |  |
| ^a^ Data are number of children with data (N), and median (interquartile range) or mean ± standard deviation or number (%). Numbers do not add up due to missing data.  ^b^ Refers to rehabilitation phase during inpatient therapeutic care. | | | |
