# Supplementary figures and images for: Weight and mid-upper arm circumference gain velocities during treatment of young children with severe acute malnutrition, a prospective study in Uganda
Source: BMC Nutr. 2021 Jun 18;7:26. doi: 10.1186/s40795-021-00428-0 (PMC8212498; doi:10.1186/s40795-021-00428-0)

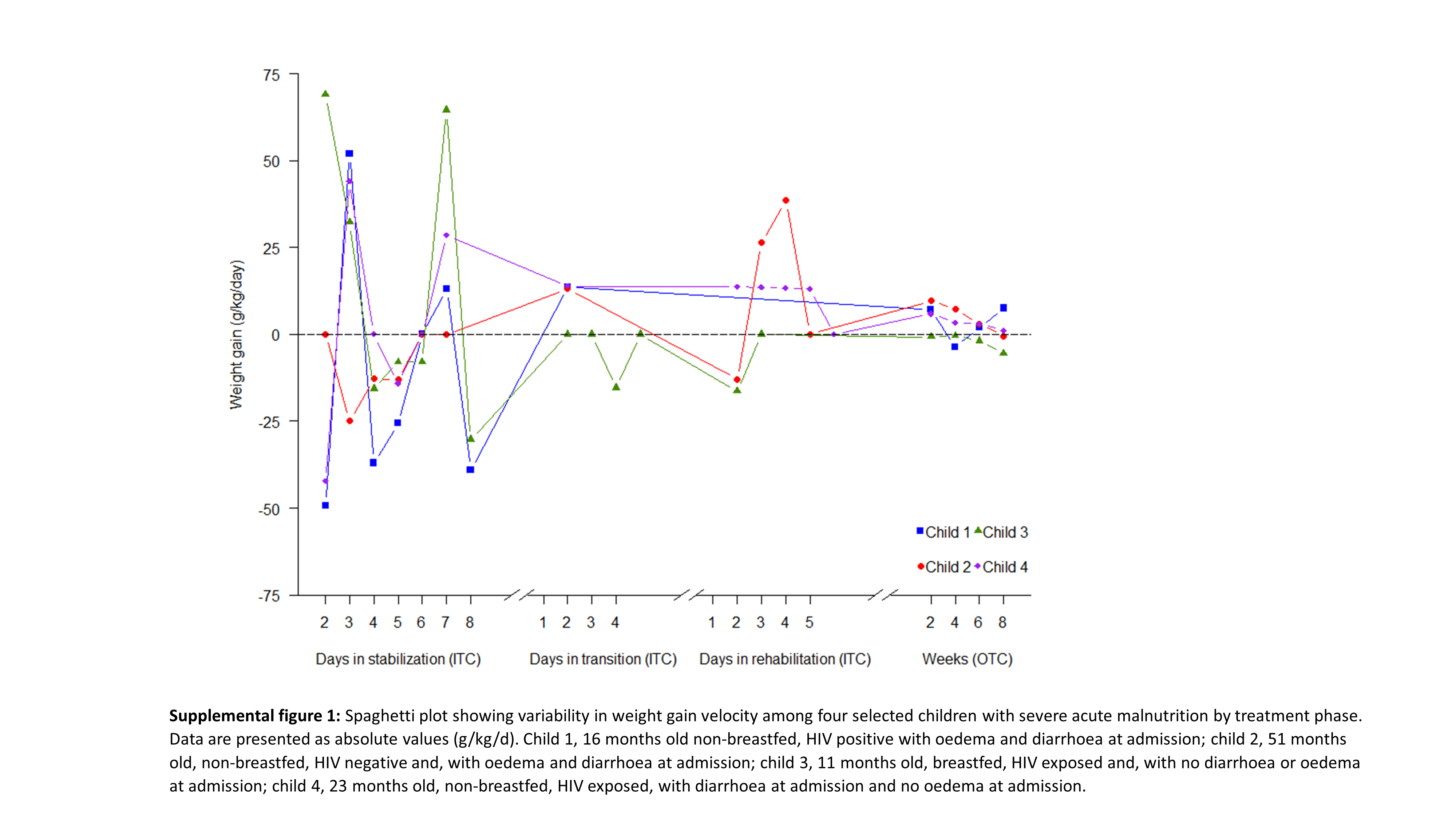

Supplement: Supplementary file 2 — Additional file 2: Supplemental Figure 1. Spaghetti plot showing variability in weight gain velocity among four selected children with severe acute malnutrition by treatment phase. Data are presented as absolute values (g/kg/d). Child 1, 16 months old non-breastfed, HIV positive with oedema and diarrhoea at admission; child 2, 51 months old, non-breastfed, HIV negative and, with oedema and diarrhoea at admission; child 3, 11 months old, breastfed, HIV exposed and, with no diarrhoea or oedema at admission; child 4, 23 months old, non-breastfed, HIV exposed, with diarrhoea at admission and no oedema at admission. [file 40795_2021_428_MOESM2_ESM.tif]

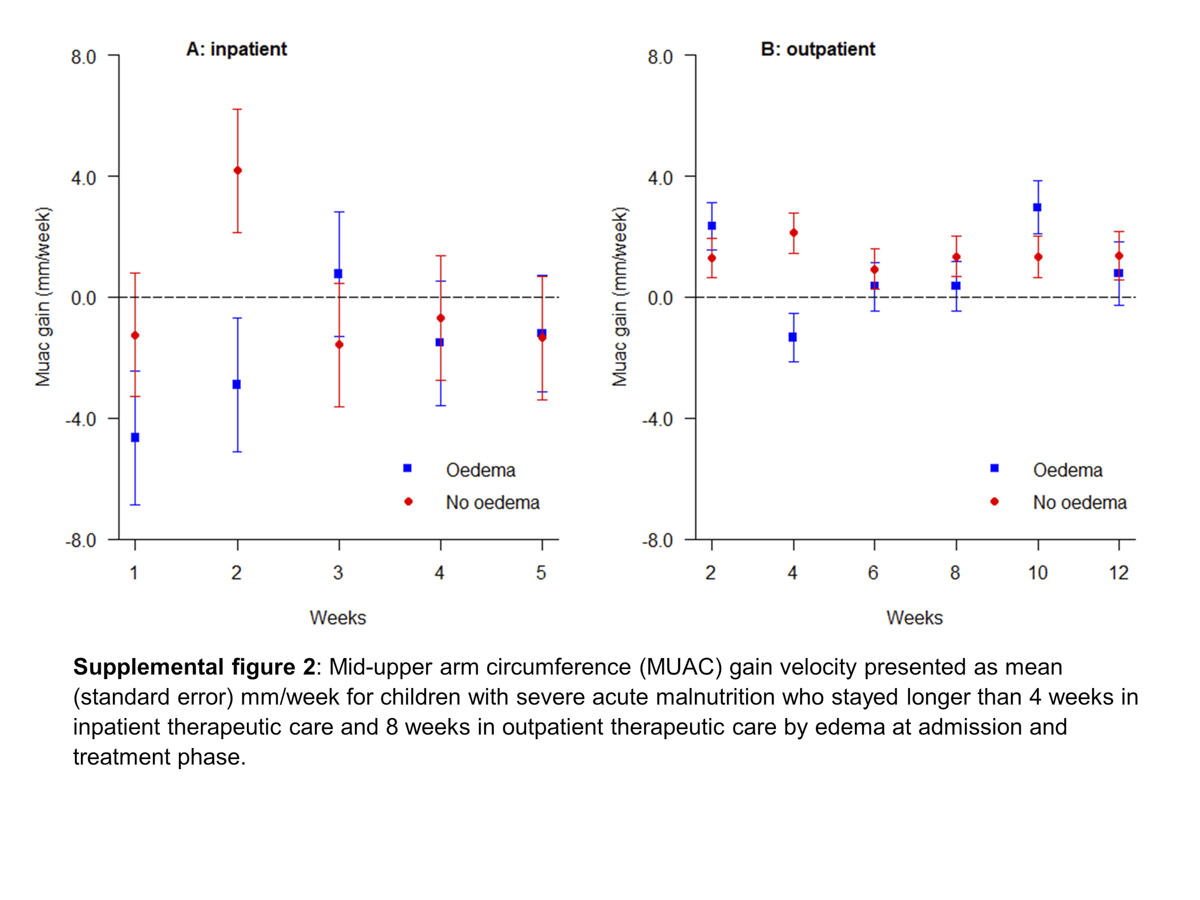

Supplement: Supplementary file 3 — Additional file 3: Supplemental Figure 2. Mid-upper arm circumference (MUAC) gain velocity presented as mean (standard error) mm/week for children with severe acute malnutrition who stayed longer than 4 weeks in inpatient therapeutic care and 8 weeks in outpatient therapeutic care by edema at admission and treatment phase. [file 40795_2021_428_MOESM3_ESM.tif]

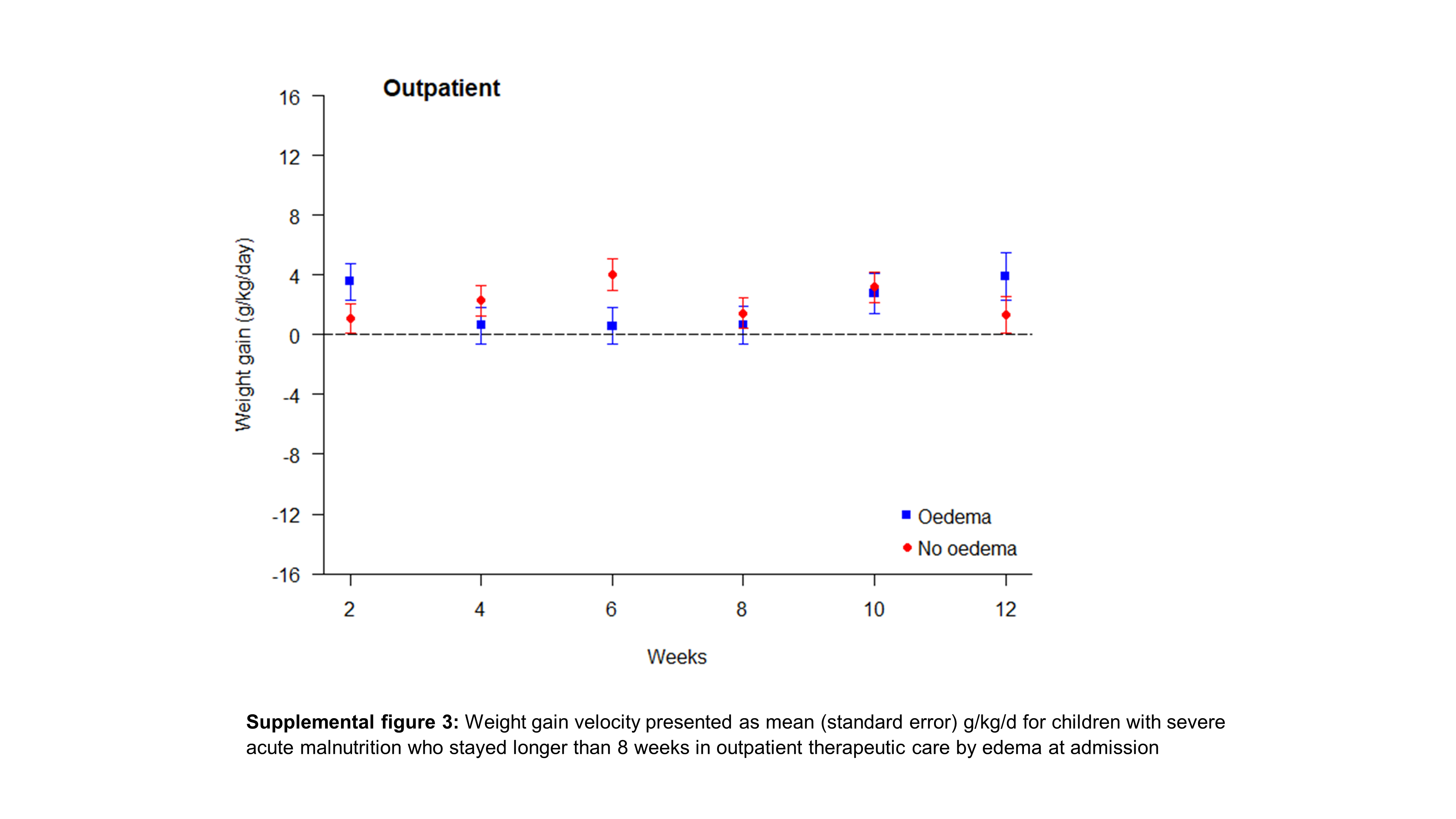

Supplement: Supplementary file 4 — Additional file 4: Supplemental Figure 3. Weight gain velocity presented as mean (standard error) g/kg/d for children with severe acute malnutrition who stayed longer than 8 weeks in outpatient therapeutic care by edema at admission. [file 40795_2021_428_MOESM4_ESM.tif]
